# Aromatic Dimer Dehydrogenases from *Novosphingobium aromaticivorans* Reduce Monoaromatic Diketones

**DOI:** 10.1128/AEM.01742-21

**Published:** 2021-11-24

**Authors:** Alexandra M. Linz, Yanjun Ma, Jose M. Perez, Kevin S. Myers, Wayne S. Kontur, Daniel R. Noguera, Timothy J. Donohue

**Affiliations:** a DOE Great Lakes Bioenergy Research Center, Univ. of Wisconsin-Madison, Madison, Wisconsin, USA; b Wisconsin Energy Institute, Univ. of Wisconsin-Madison, Madison, Wisconsin, USA; c Department of Civil and Environmental Engineering, Univ. of Wisconsin-Madison, Madison, Wisconsin, USA; d Department of Bacteriology, Univ. of Wisconsin-Madison, Madison, Wisconsin, USA; Shanghai Jiao Tong University

**Keywords:** Lignin, aromatic metabolism, *Novosphingobium*, sphingomonads, ketone reduction, aromatic dehydrogenases

## Abstract

Lignin is a potential source of valuable chemicals, but its chemical depolymerization results in a heterogeneous mixture of aromatics and other products. Microbes could valorize depolymerized lignin by converting multiple substrates into one or a small number of products. In this study, we describe the ability of Novosphingobium aromaticivorans to metabolize 1-(4-hydroxy-3-methoxyphenyl)propane-1,2-dione (G-diketone), an aromatic Hibbert diketone that is produced during formic acid-catalyzed lignin depolymerization. By assaying genome-wide transcript levels from N. aromaticivorans during growth on G-diketone and other chemically-related aromatics, we hypothesized that the Lig dehydrogenases, previously characterized as oxidizing β-O-4 linkages in aromatic dimers, were involved in G-diketone metabolism by N. aromaticivorans. Using purified N. aromaticivorans Lig dehydrogenases, we found that LigL, LigN, and LigD each reduced the Cα ketone of G-diketone *in vitro* but with different substrate specificities and rates. Furthermore, LigL, but not LigN or LigD, also reduced the Cα ketone of 2-hydroxy-1-(4-hydroxy-3-methoxyphenyl)propan-1-one (GP-1) *in vitro*, a derivative of G-diketone with the Cβ ketone reduced, when GP-1 was provided as a substrate. The newly identified activity of these Lig dehydrogenases expands the potential range of substrates utilized by N. aromaticivorans beyond what has been previously recognized. This is beneficial both for metabolizing a wide range of natural and non-native depolymerized lignin substrates and for engineering microbes and enzymes that are active with a broader range of aromatic compounds.

**IMPORTANCE** Lignin is a major plant polymer composed of aromatic units that have value as chemicals. However, the structure and composition of lignin have made it difficult to use this polymer as a renewable source of industrial chemicals. Bacteria like Novosphingobium aromaticivorans have the potential to make chemicals from lignin not only because of their natural ability to metabolize a variety of aromatics but also because there are established protocols to engineer N. aromaticivorans strains to funnel lignin-derived aromatics into valuable products. In this work, we report a newly discovered activity of previously characterized dehydrogenase enzymes with a chemically modified by-product of lignin depolymerization. We propose that the activity of *N. aromaticivorans* enzymes with both native lignin aromatics and those produced by chemical depolymerization will expand opportunities for producing industrial chemicals from the heterogenous components of this abundant plant polymer.

## INTRODUCTION

It is estimated that approximately 30% of organic carbon in the biosphere is comprised of lignin (1). Although lignin is abundant, the heterogeneous structure and chemical composition of this aromatic polymer has prevented its widespread use as a source of products for industrial applications (2). Currently, only a small fraction of available lignin is converted into chemicals, leaving the majority of the material either burned for energy or unused as a renewable source of products (3). Overcoming the inherent barriers to valorizing lignin would provide a renewable source for many chemicals currently produced from nonrenewable resources (4). We seek to understand bacterial aromatic metabolism to biologically convert deconstructed lignin into valuable chemical products.

Guaiacyl (G)- and syringyl (S)-phenylpropanoids are the most abundant monoaromatics in the lignin polymer, with hydroxylbenzoyl (H)-aromatics as common pendent groups (5). Consequently, all existing chemical methods to depolymerize lignin produce mixtures of G-, S- and H-aromatic monomers, along with dimers or oligomers containing combinations of these aromatic units (6). The heterogeneity of depolymerized lignin presents a challenge to the production of single lignin-derived products, but this could potentially be achieved using microbes to funnel diverse lignin-derived aromatic compounds through a central metabolic pathway (7, 8). We study the ability of Novosphingobium aromaticivorans DSM12444 (formerly Sphingomonas aromaticivorans F199) (9), an alphaproteobacterium capable of metabolizing a diverse array of aromatic compounds, to convert depolymerized lignin aromatics into potentially valuable products (10). N. aromaticivorans is genetically tractable and degrades many aromatic compounds completely and quickly, making it an excellent organism for studying the metabolism of lignin-derived products (11, 12). N. aromaticivorans has several advantages for studying degradation of depolymerized lignin as this bacterium can natively catabolize all three lignin monomer types (G, S, and H) (10) and can completely metabolize aromatic dimers with β-O-4 linkages (11).

In this study, we investigated how N. aromaticivorans metabolizes an aromatic G-diketone [1-(4-hydroxy-3-methoxyphenyl)propane-1,2-dione] that is a chemical by-product of both a formic acid-catalyzed lignin depolymerization process (13) and dilute acid hydrolysis of several potential lignocellulosic crops (14). Previous work has shown that *N. aromaticivorans* grew on this G-diketone, and that an engineered strain transformed it into 2-pyrone-4-6-dicarboxylic acid (PDC), a potentially valuable lignin-derived product (10). However, the enzymes that initiate G-diketone metabolism have yet to be identified, and to our knowledge, degradation of these aromatic diketones in other potential lignin-valorizing bacteria has not been described. To identify enzymes and pathways that contribute to the initial steps in metabolism of G-diketone, we measured global transcript patterns during growth on G-diketone and other G-type monoaromatics. Based on these data, we hypothesized that the enzymes LigLNDO, which encode pyridine nucleotide-dependent dehydrogenases that initiate degradation of β-O-4 linked aromatic dimers in the closely related *Sphingobium* sp. SYK-6 (15), begin the process of metabolizing G-diketone by reducing one or both of its ketone groups. To test this hypothesis, we purified LigL, LigN, and LigD and monitored the reduction of G-diketone *in vitro* using NADH as a cofactor. The results of these experiments reveal the initial steps in G-diketone reduction and how these products enter the known aromatic metabolism pathways of *N. aromaticivorans*, demonstrate an alternative reductive function for dehydrogenases previously proposed to oxidize aromatic β-O-4 linked dimers, and illustrate the potential for microbial conversion of non-native products of chemical lignin depolymerization to valuable chemicals.

## RESULTS

### Extracellular products are transiently accumulated during G-diketone utilization.

We previously demonstrated that N. aromaticivorans can grow on a mixture of either G-diketone or 1-(4-hydroxy-3,4-dimethoxyphenyl)propane-1,2-dione (S-diketone) and glucose, and that these compounds were each converted to PDC when an engineered N. aromaticivorans strain was grown in their presence (10). This finding predicted that both of these diketones were metabolized through the central pathway for aromatic metabolism (10). However, the upper pathways and enzymes that initiate transformation of aromatic diketones to central intermediates have yet to be described. We focused this study on dissecting the early steps in metabolism of G-diketone, which was the diketone with the highest conversion yield to PDC (10).

**FIG 1:**
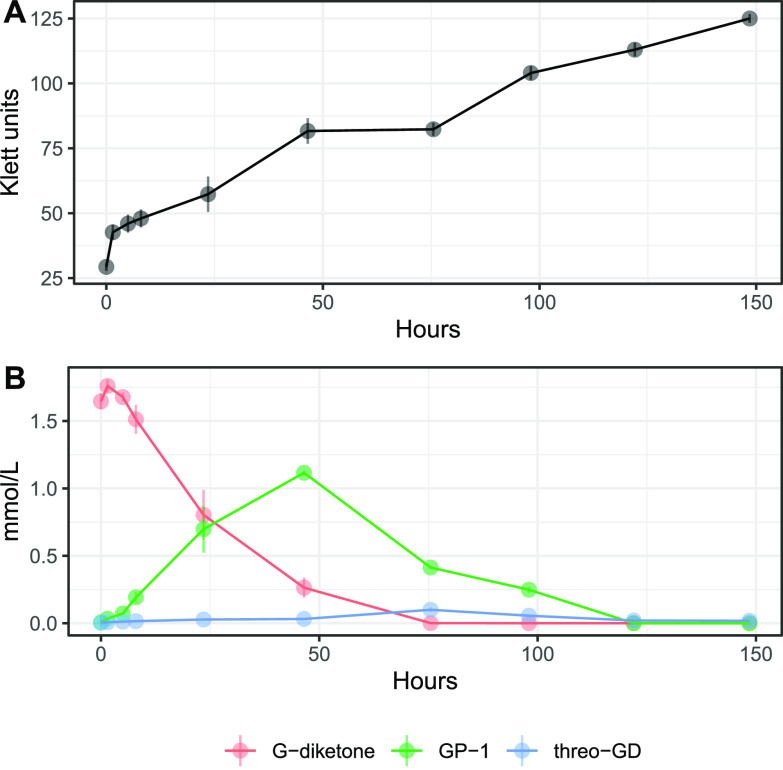
Growth and metabolism of *N. aromaticivorans* DSM12444, 12444ΔSacB strain during growth on G-diketone and glucose. Triplicate cultures were grown in SMB with 0.5 g/liter glucose and 0.418 g/liter G-diketone. In each panel, error bars represent the standard deviation. (A) Increases in *N. aromaticivorans* cell density as monitored by Klett colorimeter units. (B) Extracellular concentrations of G-diketone, GP-1, and threo-GD identified and quantified via HPLC-MS and HPLC-UV (Fig. S1, see text).

We grew N. aromaticivorans cells in media containing both G-diketone and glucose to obtain sufficient biomass for our studies. Under these conditions, high-pressure liquid chromatography (HPLC) analyses of the extracellular fractions showed a time-dependent disappearance of G-diketone from the culture media, as expected if it is imported and metabolized by N. aromaticivorans (Fig. 1). Concomitant with the disappearance of G-diketone from the media, we observed in HPLC chromatograms the transient accumulation of other extracellular compounds (Fig. S1 in the supplemental material). To investigate whether these compounds were products from reduction of the side chain in G-diketone, we compared them to authentic standards of 2-hydroxy-1-(4-hydroxy-3-methoxyphenyl)propan-1-one (GP-1), a potential derivative of G-diketone with the Cβ ketone reduced (Table S1), and threo-1-(4-hydroxy-3-methoxyphenyl)propane-1,2-diol (threo-GD), a potential derivative with both ketones reduced (Table S1). These were the only two commercially available G-diketone derivatives with a reduced side chain. Both of these standards matched the retention time and the UV-Vis spectrum of transiently accumulated metabolites (Fig. S1), allowing us to perform a preliminary quantification of some of the extracellular compounds (Fig. 1B). We considered these quantifications to be preliminary because we lacked authentic standards of 1-hydroxy-1-(4-hydroxy-3-methoxyphenyl)propan-2-one (GP-2), a GP-1 isomer with the Cα ketone reduced instead of the Cβ ketone (Table S1), and erythro-GD, the stereoisomer of threo-GD, both of which could co-elute with GP-1 or threo-GD in the HPLC. With approximately 65% of the original G-diketone apparently converted to an extracellular product in which one ketone is reduced, this preliminary quantification suggested that side chain reduction was an early step in the metabolism of G-diketone by *N. aromaticivorans*. Given the uncertainties of isomer coelutions with our HPLC method, we also analyzed by gas chromatography-mass spectrometry (GC-MS) the supernatant from samples taken halfway through the duration of the growth experiment (75.5 h). These samples and G-diketone, GP-1, and threo-GD standards were derivatized prior to GC-MS analysis (see methods). The GC chromatograms showed the presence of multiple species, and MS spectra comparison suggested the presence of GP-1 and threo-GD in these samples (Fig. S2). Furthermore, an additional GC species had an MS spectrum matching the previously published spectrum of GP-2 (14), potentially indicating that this isomer of GP-1 also accumulated in the media of G-diketone grown cells. Another species with an identical MS spectrum to threo-GD but different retention time in the GC chromatogram was observed (Fig. S2), leading us to hypothesize that this compound corresponds to the erythro-GD isomer. Thus, the identity of the extracellular materials supported the hypothesis that the transformation of G-diketone by *N. aromaticivorans* proceeded via reduction of the side chain ketones.

In growth experiments with GP-1 as the aromatic substrate, we also observed low extracellular levels of vanillin and vanillic acid (Table S2 in the supplemental material), suggesting they are products of cellular metabolism of this compound. Extracellular vanillin and vanillic acid were detected in growth experiments in which G-diketone was the aromatic carbon source (Table S2), but we cannot conclude they are derived from cellular metabolism of this carbon source since these two compounds were present at low-levels in the custom-synthesized G-diketone (Table S3).

### Genome scale changes in transcript abundance during growth in the presence of G-diketone and other G-type aromatics.

To identify candidates for gene products involved in metabolism of G-diketone, we compared transcript abundances between N. aromaticivorans cells growing in the presence of glucose alone, or with G-diketone or other individual G-type aromatics such as protocatechuic acid (PCA), vanillic acid, vanillin, GP-1, or ferulic acid (Data set S1). We found that the abundance of several hundred transcripts was altered in cells growing in the presence of G-diketone and each of the other G-type aromatics (Fig. S3) when compared with cells grown in the presence of glucose as a sole organic carbon source. Additional differences in transcript abundance were observed between cultures grown in the presence of G-diketone or GP-1 and the other G aromatics (Fig. S3).

**FIG 2:**
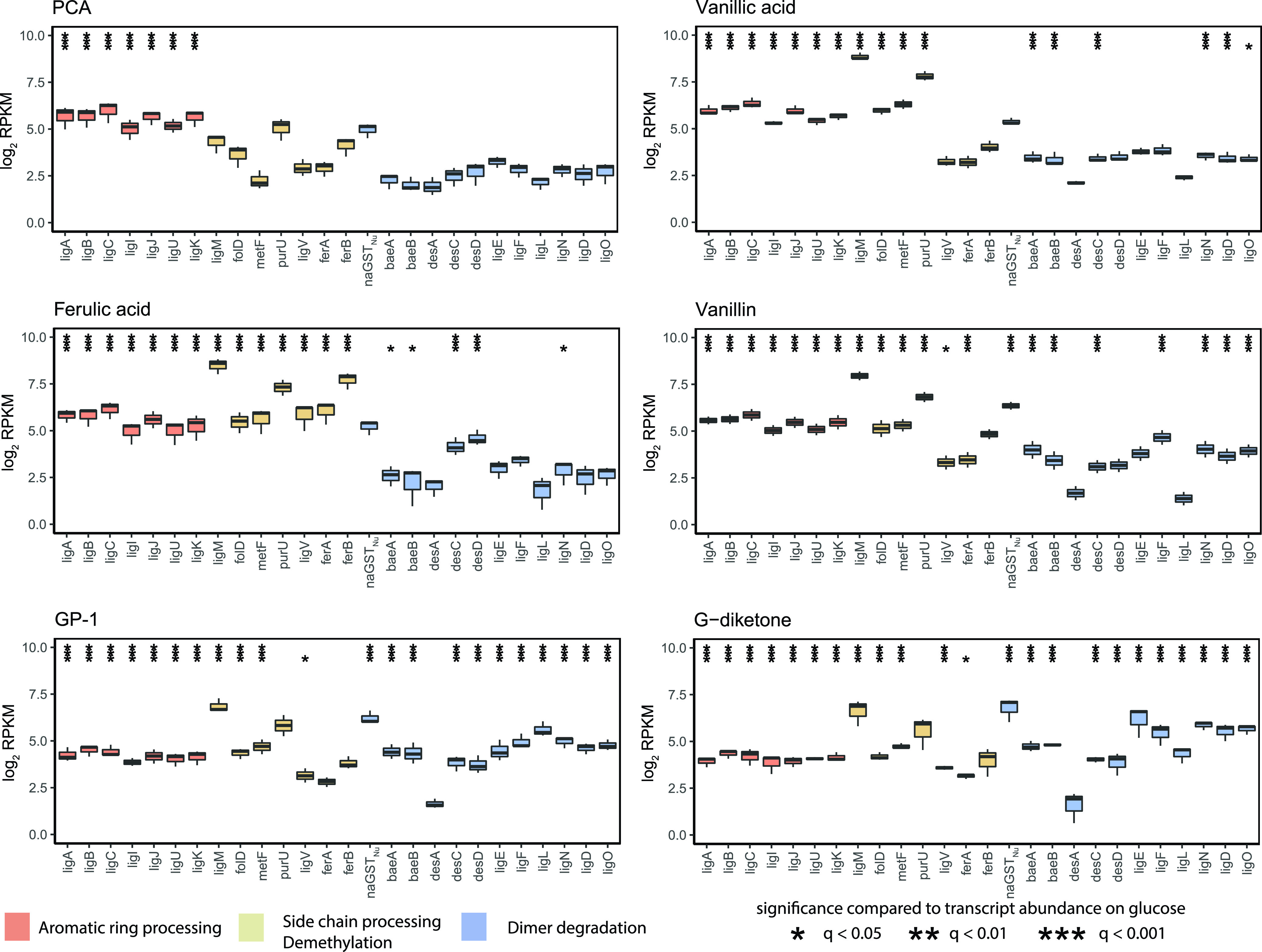
Changes in transcript abundance for indicated genes when cells are grown in the presence of G-type aromatics. Each plot displays the log_2_ fold change in reads per kilobase million (RPKM) compared with glucose-grown *N. aromaticivorans* cells showing genes identified as encoding enzymes involved in aromatic metabolism. Black stars above a transcript with a significant change in levels (*q < 0.05, **q < 0.01, ***q < 0.001) compared with cells grown in the presence of glucose alone. Bars in each panel are colored to denote steps in aromatic metabolism that gene products are known to function (dimer degradation, aromatic ring processing, side chain processing/demethylation).

Because the number of transcripts with different abundance levels in these comparisons was in the hundreds (Fig. S3, Data set S1), we focused on genes encoding enzymes known or predicted to participate in aromatic metabolism by N. aromaticivorans (Fig. 2). Trends in transcript abundance for some of these genes were as expected; for example, *ligAB*, which encodes a ring-opening dioxygenase, had increased transcript abundance in the presence of all tested aromatics compared with glucose-grown cells, while *ferAB*, which encode enzymes that act on the side chain of ferulic acid, were the most differentially expressed in the presence of ferulic acid (Fig. 2).

These data also showed that many genes encoding enzymes involved in dimer degradation were more abundant in the presence of G-diketone or GP-1 (Fig. 2), even though these are monoaromatic substrates. These differently expressed transcripts included those from genes encoding NAD-dependent dehydrogenases (*ligL*, *ligN*, *ligD*, *ligO*) (15, 16), glutathione S-transferases (*ligE*, *ligF*, *baeAB*) (17), and a glutathione lyase (*NaGST_Nu_*) (11) that are known or predicted to be involved in breaking the β-O-4 aromatic linkage in lignin and that are increased in transcript abundance in the presence of the model dimeric β-O-4 compound guaiacyl-glycerol-β-guaiacyl ether (GGE) (11) (Fig. S4).

### *In vitro* activity of Lig dehydrogenases with G-diketone.

Based on the significant increase in transcript abundance and their known ability to oxidize a hydroxyl moiety to a ketone during GGE metabolism (11), we hypothesized a potential role of the Lig dehydrogenases (LigL, LigN, LigD, LigO), which are known to catalyze reversible reactions (15) in the reduction of G-diketone. We successfully purified three recombinant Lig dehydrogenases (LigL, LigN, and LigD) and tested their activity *in vitro.* We were not able to obtain a recombinant LigO protein, so no *in vitro* assays were performed with this enzyme.

**FIG 3:**
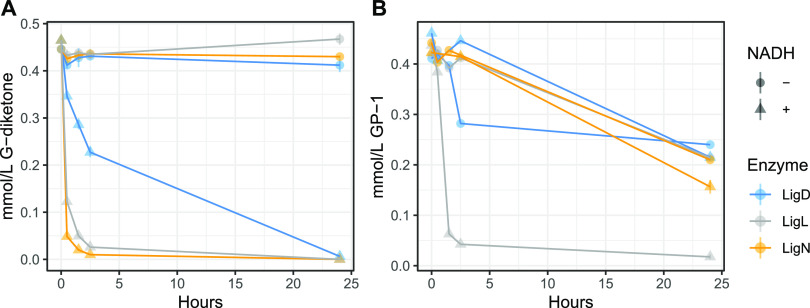
Time-dependent loss of G-diketone and GP-1 *in vitro* when incubated with recombinant LigL, LigN, and LigD, with and without NADH. 0.5 mmol/liter of G-diketone (A) or GP-1 (B) was incubated with each in enzyme with and without 2 mmol/liter NADH. Addition of NADH initiated the reaction. Samples were incubated in the dark at 30°C. Concentrations of G-diketone and GP-1 was measured using HPLC-MS.

**FIG 4:**
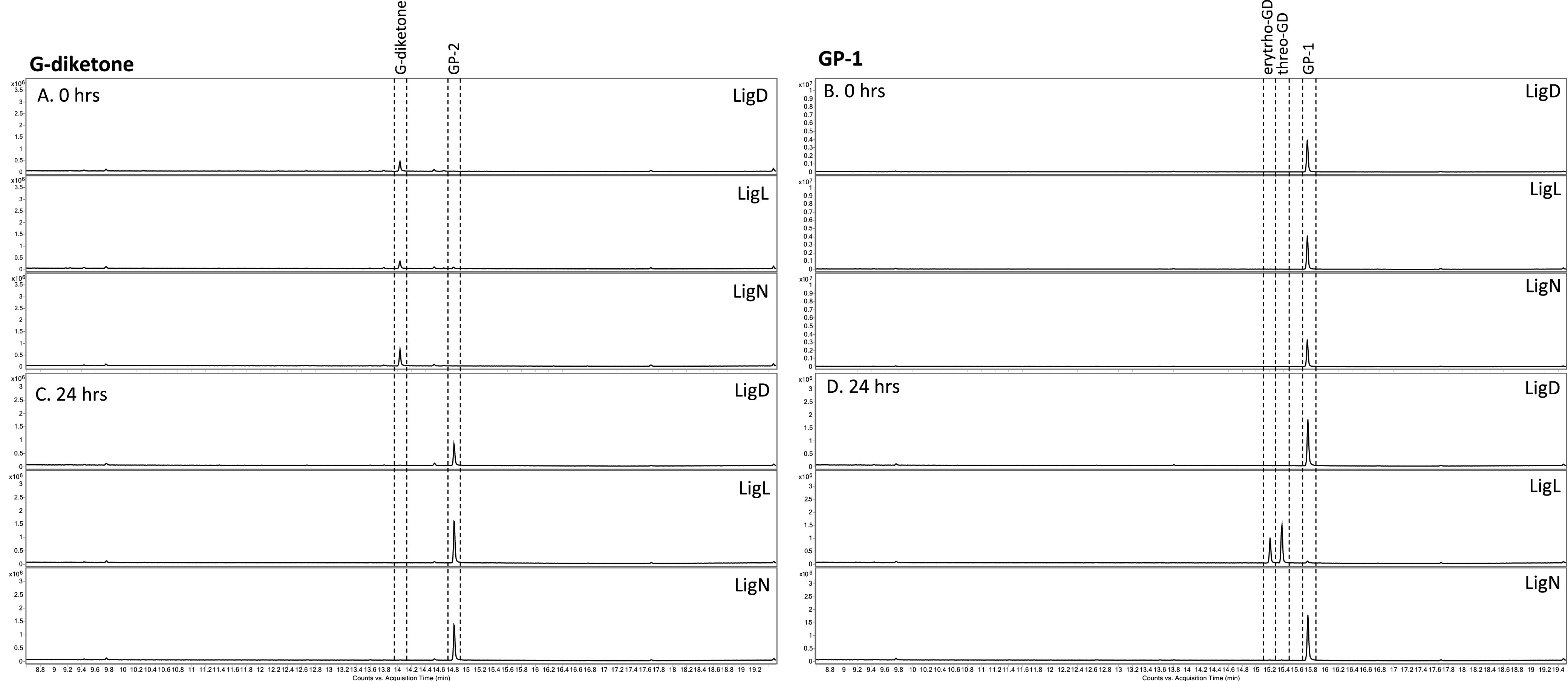
GC-MS analysis of derivatized aromatic substrates and *in vitro* reaction products of individual LigLND dehydrogenases with G-diketone and GP-1. GC-MS analysis of derivatized aromatic substrates and enzyme reaction products after indicated Lig dehydrogenases were incubated for 24 h at 30°C with the G-diketone and NADH (A, C) or GP-1 and NADH (B, D).

When testing recombinant LigL, LigN, and LigD for activity with G-diketone *in vitro*, we found that this aromatic compound was transformed in a time-dependent manner (Fig. 3). The time-dependent transformation of G-diketone required the presence of NADH (Fig. 3), suggesting that NADH is a cofactor for this activity with all three dehydrogenases, and consequently, that the transformation of G-diketone was a reduction. To better understand the activity of these Lig dehydrogenases with G-diketone, we analyzed the aromatic products of the *in vitro* reactions by GC-MS (Fig. 4). After 24 h of incubation with G-diketone and NADH, GP-2 (Table S1) was identified as the product of reactions with all three Lig dehydrogenases and G-diketone was completely transformed (Fig. 4). GP-1 was not stable in the 24-h incubations with the Lig dehydrogenases in the presence or absence of NADH (Fig. 3B), but when the products of GP-1 transformation were assayed with GC-MS, both threo- and erythro-GD were detected only in experiments with LigL (Fig. 4). These results demonstrated that LigL, LigN and LigD each have the ability to reduce the Cα ketone of G-diketone when NADH is provided as a source of reducing power, and that LigL is additionally capable of NADH-dependent reduction of the Cα ketone when the Cβ ketone is already reduced, as is the case in GP-1.

**FIG 5:**
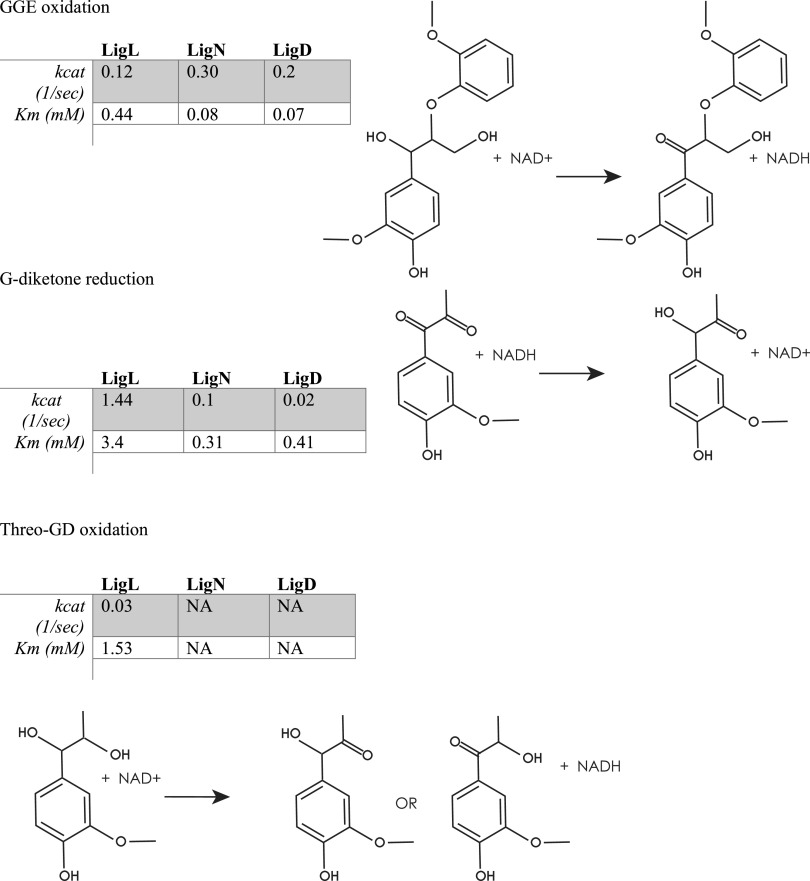
Kinetic parameters of LigL, LigN and LigD dehydrogenases with indicated aromatic substrates. Shown are the measured K_cat_ and apparent K_m_ using recombinant LigL, LigN, and LigD enzymes with the indicated aromatic substrates and either NADH (G-diketone) or NAD^+^ (GGE, GD) as a cofactor.

Since homologues of these enzymes have been studied for their NAD-dependent dehydrogenase activity on β-O-4 linked aromatic dimers (18), we measured the kinetic parameters of these recombinant Lig proteins in their oxidative and reductive directions by spectrophotometric measurement of NAD+ reduction when incubated with GGE, and of NADH oxidation when incubated with G-diketone, respectively (Fig. 5, Fig. S5 in the supplemental material). Recombinant LigL, LigN, and LigD showed relatively similar turnover frequencies (k_cat_) and Michaelis-Menten half-saturation constants (K_m_) when incubated with GGE and NAD+ (Fig. 5), as expected given previous reports of the kinetics of their homologues from *Sphingobium* SYK-6 with this substrate (18). However, the k_cat_ value for LigL with G-diketone (1.44 s^−1^) was over 10-fold greater than the k_cat_ value for the same enzyme with GGE (0.12 s^−1^). We also found that LigN and LigD both had significantly lower k_cat_ values for G-diketone than GGE (0.1 and 0.02 s^−1^ for G-diketone reduction, 0.3 and 0.2 s^−1^ for GGE oxidation, respectively) (Fig. 5). This finding suggests that LigL has a faster turnover with G-diketone than with GGE compared with LigN and LigD. For LigD, we found that its k_cat_ value with G-diketone was 10-fold lower (0.02 s^−1^) than the k_cat_ value with GGE (0.2 s^−1^), suggesting that LigD acts more rapidly on GGE than on G-diketone.

We also attempted to measure kinetic parameters of LigL with GP-1 and NADH as the substrates since this enzyme showed reductive activity with GP-1 (Fig. 4), but absorbance of a putative reaction product at 340 nm interfered with our ability to monitor NADH oxidation. The presence of a reaction product is consistent with the observed disappearance of GP-1 in the *in vitro* experiments (Fig. 3B). However, when LigL was incubated with threo-GD and NAD+, reduction of NAD+ was observed with a relatively low k_cat_ value (0.03 s^−1^), although we were unable to identify a product (Fig. 5). In addition, no detectable reduction of NAD+ was observed when LigN or LigD were incubated with threo-GD (Fig. 5).

### Genetic analysis of the role of Lig aromatic dehydrogenases in G-diketone utilization.

The results of the above *in vitro* enzyme assays indicate that LigL, LigN, and LigD can each reduce G-diketone to GP-2. If each of the Lig dehydrogenases can contribute to G-diketone metabolism *in vivo*, we predict that loss of any single Lig dehydrogenase would not have a significant impact on the growth or the production of microbial biomass of N. aromaticivorans in the presence of this aromatic compound. To test this hypothesis, we created strains with individual in-frame deletions of *ligL*, *ligN*, *ligD*, and *ligO* and tested their growth in media containing either glucose alone or glucose and G-diketone. Of the four individual mutants, none showed any significant defects in either growth rate or total biomass produced when grown on glucose as the sole carbon source or on glucose plus G-diketone (Fig. S6 in the supplemental material). These data show that none of these individual dehydrogenases is required for growth in media containing glucose and G-diketone, and they predict that each enzyme catalyzes sufficient reduction of this aromatic to support growth of the mutants at similar rates as the wild-type strain.

## DISCUSSION

Microbial funneling of the products of depolymerized lignin into valuable compounds has the potential to overcome an existing challenge caused by the heterogeneous chemical composition of this polymer. Unlike many abiotic methods, microbes can simultaneously convert mixtures of aromatic monomers to one or a few desired products, either naturally or when engineered (10, 12). Several bacteria are being developed as potential chassis organisms for microbial funneling of depolymerized lignin. Sphingomonad bacteria such as *N. aromaticivorans* and *Sphingobium* SYK-6 have the native ability to cleave β-O-4 ether linkages in lignin-derived aromatic dimers (16) and can be engineered to metabolize a wide range of substrates to produce a valuable compound such as PDC (10, 12). Similarly, the gammaproteobacterium Pseudomonas putida can either naturally produce or be engineered to produce valuable chemicals from one or more aromatic substrates (19–21). Other potential bacterial products of depolymerized lignin include *cis-cis* muconic acid, lipids, or polyhydroxyalkonates (22–26).

The success of microbial funneling is likely dependent on both the organism and the lignin depolymerization method. Some microbes may be better suited to funnel both native and chemically-modified products of lignin depolymerization, either before or after the addition of metabolic functions from another host. In this study, we examined how N. aromaticivorans consumes G-diketone, a phenylpropanone that is abundant in the products formed from a formic acid-induced lignin depolymerization method (13) and is detected in the products of dilute acid hydrolysis of several potential lignocellulosic biofuel feedstocks (14). This G-diketone belongs to a group of compounds known as Hibbert ketones (14), for which information on microbial degradation pathways is lacking. *N. aromaticivorans* is the only bacterium tested to date with the ability to catabolize these diketones. In this work, we identified enzymes that reduce the side chain ketone moieties of G-diketone. Below, we discuss the implications of our findings on the enzymes involved in the process of G-diketone degradation by N. aromaticivorans.

### Previously characterized Lig dehydrogenases reduce G-diketone.

Based on genome-wide transcript analysis of cells grown in the presence of G-diketone, we hypothesized that the known aromatic dehydrogenases LigLNDO played a role in G-diketone metabolism, possibly by reducing one or both ketones on the aromatic side chain. To test this hypothesis, we compared enzyme activity for recombinant LigL, LigN, and LigD incubated with G-diketone or GGE, a model aromatic dimer. *In vitro* enzyme assays confirmed that LigL, LigN, and LigD were all able to reduce the Cα ketone of G-diketone in a reaction that required NADH as a source of reductant to produce GP-2. Each of these Lig dehydrogenases bound G-diketone and GGE with a similar affinity, since the Michaelis-Menten constants for the monoaromatic and dimeric substrates were comparable under identical reaction conditions (Fig. 5). This predicts that the presence or absence of a second aromatic ring does not make a major contribution to binding of this substrate. This indicates that Lig enzymes analyzed in the past may have a broader role in the metabolism of biologically derived aromatics as well as those produced during chemical treatment of lignin and other aromatic-containing substrates. Indeed, our experiments show that N. aromaticivorans LigL, LigN and LigD are each able to oxidize the side chain of an aromatic dimer like GGE and reduce the Cα-side chain ketone in G-diketone to generate GP-2, and that LigL also reduces the Cα carbon of GP-1 to produce racemic GD (Fig. 4).

We found that LigN and LigD had higher turnover frequencies when oxidizing the Cα bond in the aromatic dimer GGE compared with reducing the Cα ketone in G-diketone (Fig. 5). In contrast, LigL had a higher turnover frequency when reducing the Cα ketone in G-diketone than when oxidizing the Cα position of GGE (Fig. 5). These results predict there may be active site differences in these three aromatic dehydrogenases that impact their ability to oxidize or reduce individual substrates. In addition, we found that LigL was able to reduce the Cα ketone to form GD when provided GP-1 as a substrate, while LigN and LigD could not reduce GP-1 (Fig. 5). This suggests that LigL may catalyze another step in G-diketone degradation, resulting in a fully reduced side chain that is a substrate for subsequent cleavage to produce vanillin. Hibbert ketones such as GP-2 and GP-1 are known to spontaneously interconvert, so it is possible that, under cellular conditions, GP-2 can isomerize to GP-1 (14). Deletion of *ligL* did not result in a growth defect on G-diketone, so it is possible that LigO, which we were not able to purify, or another as of yet unknown enzyme is capable of reducing GP-2 to GD. Lig dehydrogenases from other sphingomonads have been found to be stereospecific (18) or stereoselective (27) for the stereoconfiguration of Cα in GGE, so it is likely that stereochemistry may play an important role in enzymatic processing of G-diketone side chain. However, sources of purified GP-2 and erythro-GD are needed to assess the relative activity of LigD, LigL, and LigN with these compounds.

### *In vivo* degradation of G-diketone.

Our *in vitro* enzyme assays predicted that G-diketone degradation is initiated by the NADH-dependent reduction of the Cα ketone, producing GP-2. When *N. aromaticivorans* was grown in the presence of G-diketone and glucose, we observed the transient extracellular accumulation of a compound with the same retention time of GP-1 (Fig. 1) but were unable to confirm whether GP-1 and GP-2 coeluted in the HPLC method used, although GC-MS analyses confirmed that both of these ketones were present in the culture media. GC-MS analyses also confirmed the presence of threo-GD and erythro-GD in culture media, supporting the concept of multiple reduction steps in the initial steps of transformation of G-diketone. Strains containing single deletions of *ligLNDO* did not have a fitness defect in the presence of G-diketone; therefore, while our *in vitro* assays suggest that these dehydrogenases can complement functions, we cannot rule out the presence of other enzymes *in vivo* that may also be able to reduce G-diketone. We also cannot conclusively demonstrate that LigL, LigN, and LigD perform the same functions *in vivo* as we observed *in vitro*.

**FIG 6:**
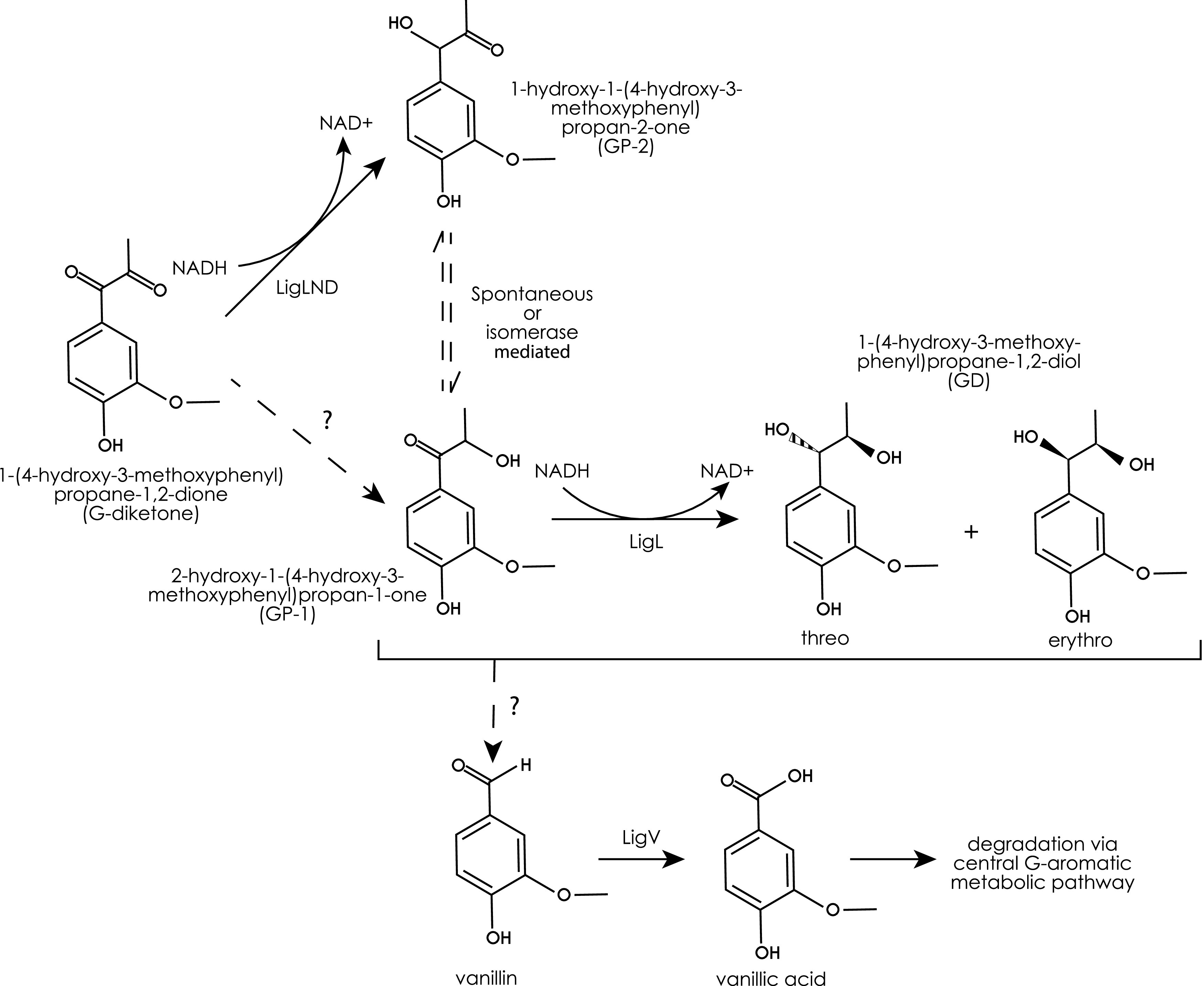
Model for G-diketone metabolism by *N. aromaticivorans.* We hypothesize that the indicated Lig dehydrogenases initiate degradation of G-diketone, reducing the Cα ketone to GP-2. GP-2 and GP-1, as Hibberts ketones, can spontaneously interconvert; the question marks indicate that we cannot rule out the existence of enzymes that produce GP-1. The figure also indicates that the LigL dehydrogenase reduced GP-2 to 1-(4-hydroxy-3-methoxyphenyl)propane-1,2-diol (GD). In this model, one or more unknown enzymes, indicated by the question mark, are used to produce vanillin from GP-1 or GD.

Based on the results of our *in vitro* analyses of these aromatic dehydrogenases, we propose a model for metabolism of G-diketone by N. aromaticivorans (Fig. 6). This model predicts that one of several Lig dehydrogenases initially reduce the Cα ketone of G-diketone to GP-2, which can then isomerize to GP-1. Alternatively, GP-1 could be produced by other enzymes not identified in this study. The model also predicts that LigL reduces GP-1 to racemic GD. Next, we propose that an as of yet uncharacterized enzyme(s) cleaves the side chain of GD, producing an unknown two-carbon product and vanillin, which is oxidized by a homologue of LigV, which is known to convert vanillin into vanillate in *Sphingobium* SYK-6 (28) (Fig. 6). Consistent with this model, transcripts derived from *N. aromaticivorans ligV* were more abundant during growth on both G-diketone and GP-1 than during growth on glucose alone (Fig. 2) (28). An alternative model predicts that GD is not an intermediate in G-diketone degradation, but a side product in a pathway in which vanillin is produced directly from GP-1 or GP-2 by as of yet unidentified enzymes. The transient extracellular accumulation of vanillin and vanillic acid when cells are grown in the presence of GP-1 (Table S2 in the supplemental material) supports a proposed *N. aromaticivorans* pathway for G-diketone metabolism in which vanillic acid is an intermediate that enters the central metabolic pathways for the degradation of G-type aromatic compounds (29). Unfortunately, our G-diketone preparations contain trace amounts of vanillin, making it difficult to make a similar conclusion about the role of vanillin as an intermediate when cells metabolize G-diketone (Table S3). We also acknowledge that our experiments do not preclude the presence of additional enzymes with redundant functions in our proposed model and are primarily based on *in vitro* data, as strains containing individual deletions of each aromatic dehydrogenases showed no fitness defect in comparison to the parent strain (Fig. S6).

### Substrate specificity of Lig pathway enzymes.

When taken together, this work expands our knowledge of the ability of *N. aromaticivorans* to metabolize and funnel a diversity of aromatic compounds into a common pathway. While this work focused on enzymes needed for metabolism of G-diketone, previous studies have shown that cells can grow on S-diketone (10). It is known that several Lig enzymes are active with G- and S-type aromatic substrates (16, 30), so it is possible that these same Lig dehydrogenases can metabolize each of these respective diketones. As enzyme redundancy is a described characteristic of *N. aromaticivorans’* dimer degradation pathway (12, 17), it is also possible that additional, undiscovered enzymes can also reduce diketones in addition to the Lig dehydrogenases. We propose that the use of multi-functional Lig enzymes may provide *N. aromaticivorans* with the flexibility to consume a broad mix of monomeric and oligomeric aromatic substrates.

There are other examples of N. aromaticivorans enzymes that can act on both aromatic monomers and dimers. NaLigF2 is a β-etherase that cleaves aromatic dimers in the presence of glutathione (16). However, based on fitness analysis of a mutant library of N. aromaticivorans, this same gene product was proposed to be a subunit of a DesCD isomerase that converts 4-carboxy-2-hydroxy-6-methoxy-6-oxohexa-2,4-dienoate (CHMOD) to (4 E)-oxalomesaconate (OMA) during syringic acid degradation (29). As predicted, mutations that inactivate *desD* resulted in a loss of growth on syringic acid and accumulation of CHMOD, and *in vitro* enzyme assays showed that purified DesCD produced OMA (29). In addition, the syringate o-demethylase DesA can also demethylate vanillic acid, albeit at a slower rate, and a second ring-opening dioxygenase, LigAB2, has been identified in N. aromaticivorans (12). Thus, it is possible that broad substrate specificity may be an underappreciated feature of N. aromaticivorans aromatic metabolism that is more common than previously thought.

The presence of multifunctional, aromatic-degrading enzymes may allow *N. aromaticivorans* to adapt to, metabolize, and persist in the presence of a wide set of aromatic substrates that it might find in nature. This enzymatic flexibility is also potentially advantageous from an industrial production standpoint, since *N. aromaticivorans* could be well suited for metabolism of both naturally occurring aromatics and those that are chemical by-products of lignin or other aromatic substrates. The relatively broad substrate specificity of *N. aromaticivorans* enzymes may also provide scaffolds for improvement in catalytic rate or utilization of aromatic substrates.

In conclusion, we coupled metabolite analyses, transcriptomics, and enzyme assays to predict and identify proteins responsible for the early steps in degradation of G-diketone by N. aromaticiovorans. These studies showed that three Lig dehydrogenases previously described for a role in oxidation of a Cα carbon in β-O-4 linked aromatic dimers reduce the Cα ketone as a first step in the metabolism of G-diketone. Based on this and other published studies, we propose that redundant multi-functional enzymes are a key feature of N. aromaticivorans*’* aromatic metabolism, allowing it to degrade diverse aromatic compounds, including ones found in nature and those generated as a by-product of chemical deconstruction of lignin. This finding expands our understanding of microbial aromatic metabolism, and it furthers our ability to identify and engineer a microbial strain capable of valorizing lignin or chemical products of its deconstruction as part of biorefinery pipeline.

## MATERIALS AND METHODS

### Analyzing growth with and metabolism of G-diketone.

N. aromaticivorans cultures were grown aerobically in Standard Mineral Broth (SMB; DSMZ Medium 1185) without tryptone or yeast extract (31), supplemented with either glucose, an aromatic substrate, or both, shaken at 200 rpm and incubated at 30°C. We measured cell density using a Klett-Summerson photoelectric colorimeter with a red filter; one Klett unit (KU) is equivalent to 8 × 10^6^ CFU/ml for *N. aromaticivorans* (11).

To assay metabolism of G-diketone, triplicate cultures of N. aromaticivorans were grown overnight with 1 g/liter d-glucose (Sigma-Aldrich, St. Louis, MO) before adding an equal volume of SMB containing 1 g/liter glucose and incubating for 1 h. We then harvested 2 ml of the culture (3075 rcf, 5 min, room temperature) and resuspended the cells into 0.5 ml fresh SMB with no added carbon source. The resuspended cells were inoculated (1% inoculation ratio by volume) into 50 ml SMB containing either 1 g/liter glucose or 0.5 g/liter glucose plus 0.418 g/liter G-diketone. One-milliliter samples of these cultures were collected as a function of time and harvested (3,075 rcf, 5 min, 4°C). The supernatants of these samples were filtered through 0.22 μm nylon syringe tip filters (Fisher Scientific, Hampton, NH), analyzed by HPLC with MS and photodiode array detectors, and GC-MS.

### RNA-Seq culture conditions.

The strain used for RNA preparation was *N. aromaticivorans* DSM12444 containing a deletion of the gene *sacB* (SARO_RS09410, Saro_1879), which conveys sucrose sensitivity (11) (Table 1). All cultures were grown in SMB with 0.5 g/liter of d-glucose plus an indicated aromatic, as cultures grown on some aromatic compounds as the sole organic substrate produced insufficient biomass for RNA analysis. Aromatic substrates used were PCA, vanillic acid, vanillin, ferulic acid (Sigma-Aldrich, St. Louis, MO), GP-1 (Key Organics, Camelford, UK), and G-diketone, synthesized as previously described (10). Amounts of aromatic substrate in the culture were normalized to have a theoretical COD equivalent to 0.5 g/liter (amount added to each culture: ferulic acid, 0.29 g/liter; vanillin, 0.28 g/liter; vanillic acid, 0.33 g/liter; G-diketone, 0.29 g/liter; GP-1, 0.27 g/liter; PCA, 0.37 g/liter [Data set S1]). The aromatic substrates were dissolved in dimethyl sulfoxide (DMSO) (5 μl per 10 ml of the final culture) prior to addition to the media. Cells grown in the presence of 1 g/liter of glucose were used as the control culture for RNA analysis.

**TABLE 1:** Strains used in this study

Strain name	Parent strain	Description
12444ΔSacB	Wild type (DSM 12444)	Sucrose sensitivity gene deleted (11)
12444ΔLigL	12444ΔSacB	Markerless deletion of *ligL*
12444ΔLigN	12444ΔSacB	Markerless deletion of *ligN*
12444ΔLigD	12444ΔSacB	Markerless deletion of *ligD*
12444ΔLigO	12444ΔSacB	Markerless deletion of *ligO*

A 25-ml overnight culture grown in the presence of SMB plus glucose provided the inoculum for all cultures used for RNA extraction. Before shifting cells to media containing an aromatic substrate, we added 25 ml of SMB plus glucose and waited 40 min to allow cells to reenter growth phase. At this time, 2-ml aliquots of this culture were harvested (6,000 rpm, 5 min, 21°C), washed in 1 ml of SMB with no added carbon source, and resuspended into 100 μl of SMB with no added carbon source. Equal volumes of this cell suspension were added to 25-ml triplicate cultures containing either glucose and an aromatic compound or only glucose as the sole carbon source at concentrations described above. These cultures were incubated overnight and then amended with 25 ml of fresh identical media to ensure logarithmic growth. After 40 min, cell growth was terminated by adding 5.7 ml of cold 95% ethanol + 5% acid phenol:chloroform to the entire 50-ml culture. Cells from each culture were harvested (6,000 rpm, 12 min, 4°C), the pellets were flash-frozen in ethanol and dry ice, and the supernatant from each culture was filtered and stored at −80°C for subsequent HPLC analysis (see above).

### RNA preparation.

Thawed cell pellets were lysed using an SDS/EDTA-based buffer at 65°C (11). Genomic DNA was removed, and RNA purified using the Qiagen RNEasy Kit (Qiagen, Hilden, Germany). An on-column DNase digestion (Qiagen, Hilden, Germany) was performed to remove remaining DNA. RNA was eluted in 50 μl nuclease-free water (Life Technologies, Carlsbad, CA) and stored at −80°C until preparation for sequencing. RNA yield was assessed using a Qubit Broad-Range RNA assay (Life Technologies, Carlsbad, CA).

### RNA sequencing and bioinformatics.

RNA-Seq library preparation and sequencing was performed at the Department of Energy Joint Genome Institute (Berkeley, CA). Libraries were created using the Illumina TruSeq Stranded Total RNA kit (Illumina, San Diego, CA) following the standard protocol, which included rRNA depletion using the RiboZero bacterial kit (Illumina, San Diego, CA). Four RNA-seq libraries were sequenced per lane on an Illumina NextSeq (Illumina, San Diego, CA) in 2 × 151 reads using the manufacturer’s standard protocol.

After sequence analysis, the paired-end FASTQ files were split into those containing forward and reverse reads, and forward read files were retained for further analysis. Sequence reads were trimmed using Trimmomatic version 0.3 (32) with the default settings except for a HEADCROP of 5, LEADING of 3, TRAILING of 3, SLIDINGWINDOW of 3:30, and MINLEN of 36. After trimming, the sequence reads were aligned to the *N. aromaticivorans* genome sequence (GenBank accession NC_007794.1) using Bowtie2 version 2.2.2 (33) with default settings except the number of mismatches was set to 1. Aligned sequence reads were mapped to gene locations using HTSeq version 0.6.0 (34) with default settings except that the “reverse” strandedness argument was used. The software edgeR version 3.26.8 (35) was used to identify significantly differentially expressed genes from pairwise analyses based on *q* values, using a Benjamini and Hochberg false discovery rate less than 0.05 as a significance threshold (35–37). Raw sequencing reads were normalized using the reads per kilobase per million mapped reads. Subsequent analyses and plots were prepared in R 3.5.1 (R Core Team, Vienna, Austria) using the packages tidyverse 1.3.0, reshape2 1.4.3, and cowplot 1.0.0.

### Construction of *N. aromaticivorans* mutants.

Strains containing individual in-frame deletions of *ligL*, *ligN*, *ligD*, and *ligO* (SARO_RS09390, SARO_RS03965, SARO_RS01025, and SARO_RS03960, respectively) were generated in a Δ*sacB* mutant of wild-type strain DSM12444 using previously described plasmids and methods (Table 2) (29). Briefly, strains of the Escherichia coli WM3064 containing nonreplicating pAK405 vectors with approximately 450 bp of DNA flanking each desired gene deletion were conjugated on solid medium in a 1:5 ratio with the *N. aromaticivorans* Δ*sacB* recipient (38). Conjugates in which the plasmid was incorporated into the genome via homologous recombination were selected by plating onto LB with kanamycin, then were grown overnight in 5-ml cultures of LB and plated onto LB with streptomycin to select for strains from which the plasmid was excised via a second round of homologous recombination. Mutants with desired gene deletions were confirmed via the ability to grow in the presence of streptomycin and inability to grow in the presence of kanamycin, as well as via PCR of genomic DNA with gene-specific primers (Table 3). Growth of confirmed deletion mutants and the Δ*sacB* parent strain was tested in 25-ml triplicate flask cultures containing 1 mmol/liter glucose + 1 mmol/liter G-diketone in SMB + 0.05% DMSO, as well as additional cultures grown in 2 mmol/liter glucose as a control.

**TABLE 2:** Plasmids used to inactivate indicated *lig* genes in this study (29)

Plasmid name	Base plasmid^*a*^	Gene deletion
pJM307	pAK405	*ligD*
pJM311	pAK405	*ligO*
pJM312	pAK405	*ligN*
pJM323	pAK405	*ligL*

a See reference 38.

**TABLE 3:** Primers for confirming *lig* deletion mutants (29)

Name	Forward primer	Reverse primer	Target
01025	TCAGGTCCACCAGTTCGCCATC	GTCTCTATCGCGTTGACCGACTGG	*ligD*
03960	ACAAGAACTTCGGCCTCTATCGTGAC	GTGAAGCTCGACGTGACCAATCG	*ligO*
03965	CGCGAACTTGGTGGTATTGTAGATGC	CGAAAAGGCGCGAGTGATCTTCTTC	*ligN*
09390	GCTATGCCGAATTTGCCCTGAC	CTGTCGGGATATGCCATCTACATCTGG	*ligL*

### *In vitro* enzyme assays.

The genes *ligL* (SARO_RS09390), *ligN* (SARO_RS03965), and *ligD* (SARO_RS01025) were amplified from *N. aromaticivorans* DSM12444 and separately cloned into plasmid pVP302K (16) containing an N-terminal His_8_ tag. The expression plasmids were transformed into E. coli B834 containing pRARE2 plasmid (Novagen, Gibbston, NJ). Protein expression was induced by growing the E. coli strains at 25°C for 25 h in ZYM-5052 autoinduction medium containing kanamycin and chloramphenicol (39). Cells were harvested by centrifugation. The resulting pellet was resuspended in lysis buffer (50 mM NaH_2_PO_4_, 100 mM NaCl, 5 mM imidazole, 10% glycerol, 0.5 mM TCEP, and 1% TritonX-100) and lysed by sonication. Following centrifugation of the lysates, the supernatant was filtered through a 0.22-μm, 33-mm diameter, polyethersulfone filter (MilliporeSigma, Burlington, MA) and passed through a gravity column packed with Ni^2+^-NTA resin (Qiagen, Hilden, Germany), washed with wash buffer (50 mM NaH_2_PO_4_, 200 mM NaCl, 25 mM imidazole, and 0.5 mM TCEP) and eluted in elution buffer (50 mM NaH_2_PO_4_, 300 mM NaCl, 500 mM imidazole, and 0.5 mM TCEP). The eluted proteins were concentrated using Amicon Ultra-15 centrifugal filter units (MilliporeSigma, Burlington, MA) and dialyzed into dialysis buffer (50 mM NaH_2_PO_4_, 100 mM NaCl, and 0.5 mM TCEP), then flash frozen and stored at −80°C until further study. Protein concentrations were determined using the Bradford method.

These purified enzymes were used for *in vitro* enzyme assays using 0.5 mM G-diketone as a potential aromatic substrate along with 1 μM enzyme with or without 2 mM NADH (Sigma-Aldrich, St. Louis, MO) as a cofactor, in a buffer containing 25 mM Tris-HCl (pH 8) and 25 mM NaCl (Sigma-Aldrich, St. Louis, MO). An additional control was run with no enzyme added to assess spontaneous degradation of G-diketone. Assays were incubated at 30°C in the dark. In some cases, concentrations of G-diketone were measured at 0, 0.5, 1.5, 2.5, and 24 h using HPLC-MS. We tested for the presence of additional reaction products by GC-MS using material from the 24-h time point.

Spectrophotometric NADH oxidation/NAD+ reduction assays were performed using LigL, LigN, and LigD in the presence of G-diketone, GP-1, GGE, or threo-GD (BioCrick Co. Ltd., Chengdu, Sichuan, China). Assay conditions were set as described above at lower concentrations to remain within accurate range of the spectrophotometer (0.005-0.2 mM substrate, 0.1 mM co-factor, and 0.1 μM enzyme (0.01 μM for LigL with G-diketone due to increased reaction velocity)), with NADH or NAD+ added immediately prior to measurement of optical density at 340 nm on an Olis DW-2000 spectrophotometer (OLIS, Inc., Athens, GA). An assay with no pyridine nucleotide cofactor was used as the reference for set of reactions. Substrate concentrations were varied to determine the kinetic parameters of each enzyme.

### Chemical analysis.

Quantitative analyses of G-diketone, GP-1, and vanillic acid were performed on a Shimadzu triple quadrupole HPLC-MS (Shimadzu model Nexera XR, HPLC-8045 MS/MS). Reverse-phase HPLC was performed using a binary gradient mobile phase consisting of Solvent A (0.2% formic acid in water) and solvent B (methanol) at a flow rate of 0.4 ml/min. The column was conditioned at 5% B, the elution program was 5% B hold 0.1 min, ramp to 20% B at 0.5 min, ramp to 30% B at 3.5 min, ramp to 50% B at 5 min, ramp to 95% B at 5 min and hold for 1.5 min to wash the column, then reset the column by returning to 5% B at 7 min and holding for 2.5 min to equilibrate the column for the next injection. The stationary phase was a Kinetex F5 column (2.6 μm pore size, 2.1 mm ID, 150 mm length, P/N: 00F-4723-AN). All compounds listed above were detected by multiple-reaction-monitoring (MRM) and quantified using the strongest MRM transition (Table S4). Threo-GD was quantified by light absorbance at 280 nm using a photodiode array detector (Shimadzu model SPD-M20A).

GC-MS was performed to detect and identify potential products of G-diketone and GP-1 metabolism. Sample aliquots (150 μl) were acidified with HCl to pH < 2, and ethyl acetate extracted (3 × 500 μl). The three extraction samples were combined, dried under a stream of N_2_ at 40°C, derivatized by the addition of 150 μl of pyridine and 150 μl of N,O-bis(trimethylsilyl)trifluoro-acetamide with trimethylchlorosilane (99:1, wt/wt, Sigma) and incubated at 70°C for 45 min. The derivatized samples were analyzed on an Agilent GC-MS (GC model 7890A, MS Model 5975C) equipped with a (5% phenyl)-methylpolysiloxane capillary column (Agilent model HP-5MS). The injection port temperature was held at 280°C and the oven temperature program was held at 80°C for 1 min, then ramped at 10°C min^−1^ to 220°C, held for 2 min, ramped at 20°C min^−1^ to 310°C, and held for 6 min. The MS used an electron impact (EI) ion source (70 eV) and a single quadrupole mass selection scanning at 2.5 Hz, from 50 to 650 *m/z*. The data was analyzed with Agilent MassHunter software suite. GC-MS spectrum and retention times for GP-1 and threo-GD were compared with authentic standards. The identity of GP-2 was confirmed by comparison with a published GC-MS spectrum (14). The identity of erythro-GD was elucidated by GC-MS spectrum comparison with the one produced by threo-GD authentic standard and the difference in retention time of threo-GD (Table S1 in the supplemental material).

### Data availability.

Raw sequencing reads are available through the DOE Joint Genome Institute Genome Portal with Project ID 1233250 (https://genome.jgi.doe.gov/portal/Novarocriptomics_FD/Novarocriptomics_FD.info.html). Processed transcriptomic data are available through NCBI GEO (accession number GSE174697). Code used in this project and other data reference in this study can be found in the GitHub repository (https://github.com/GLBRC/AromaticDiketones).
