# Supplementary material for: Aromatic Dimer Dehydrogenases from Novosphingobium aromaticivorans Reduce Monoaromatic Diketones
Source: Appl Environ Microbiol. 2021 Nov 24;87(24):e01742-21. doi: 10.1128/AEM.01742-21 (PMC8612281; doi:10.1128/AEM.01742-21)
Supplement: Supplemental file 1 — Tables S1 to S4, Fig. S1 to S6, legend to Data Set S1. Download aem.01742-21-s0001.pdf, PDF file, 0.6 MB [file aem.01742-21-s0001.pdf]

Table S1. Characteristics of G-diketone and other aromatics analyzed by chromatography

Short Name	Structure	IUPAC Name	Molecular Formula	Molecular Weight (g/mol)	Retention time in HPLC (min)	Retention time in GC (min)*
G-diketone	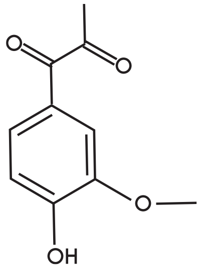	1-(4-hydroxy-3-methoxyphenyl) propane-1,2-dione	$C_{10}H_{10}O_4$	194.180	4.20	14.0
GP-1	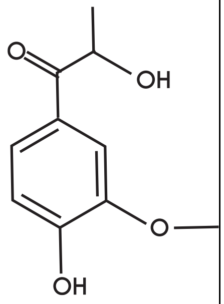	2-hydroxy-1-(4-hydroxy-3-methoxyphenyl) propan-1-one	$C_{10}H_{12}O_4$	196.200	2.86	15.8
GP-2	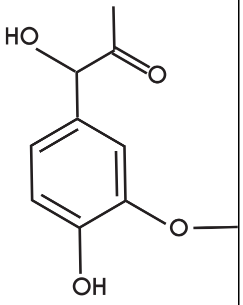	1-hydroxy-1-(4-hydroxy-3-methoxyphenyl) propan-2-one	$C_{10}H_{12}O_4$	196.200	Unknown	14.8

threo-GD	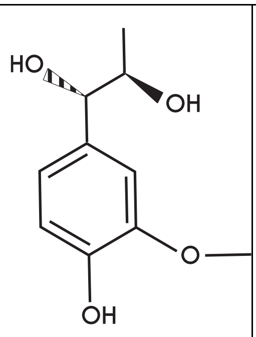	threo-1-(4-hydroxy-3-methoxyphenyl) propane-1,2-diol	$C_{10}H_{14}O_4$	198.220	2.08	15.4
erythro-GD	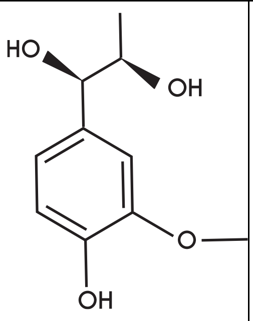	erythro-1-(4-hydroxy-3-methoxyphenyl) propane-1,2-diol	$C_{10}H_{14}O_4$	198.220	Unknown	15.2
Vanillic acid	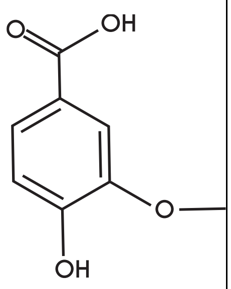	4-hydroxy-3-methoxybenzoic acid	$C_8H_8O_4$	168.150	2.54	14.4
Vanillin	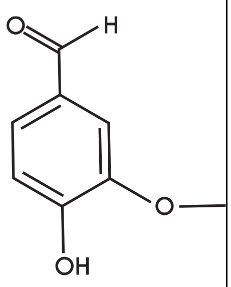	4-hydroxy-3-methoxybenzaldehyde	$C_8H_8O_3$	152.150	2.84	12.0

* Retention time in GC is for TMS derivatized compound.

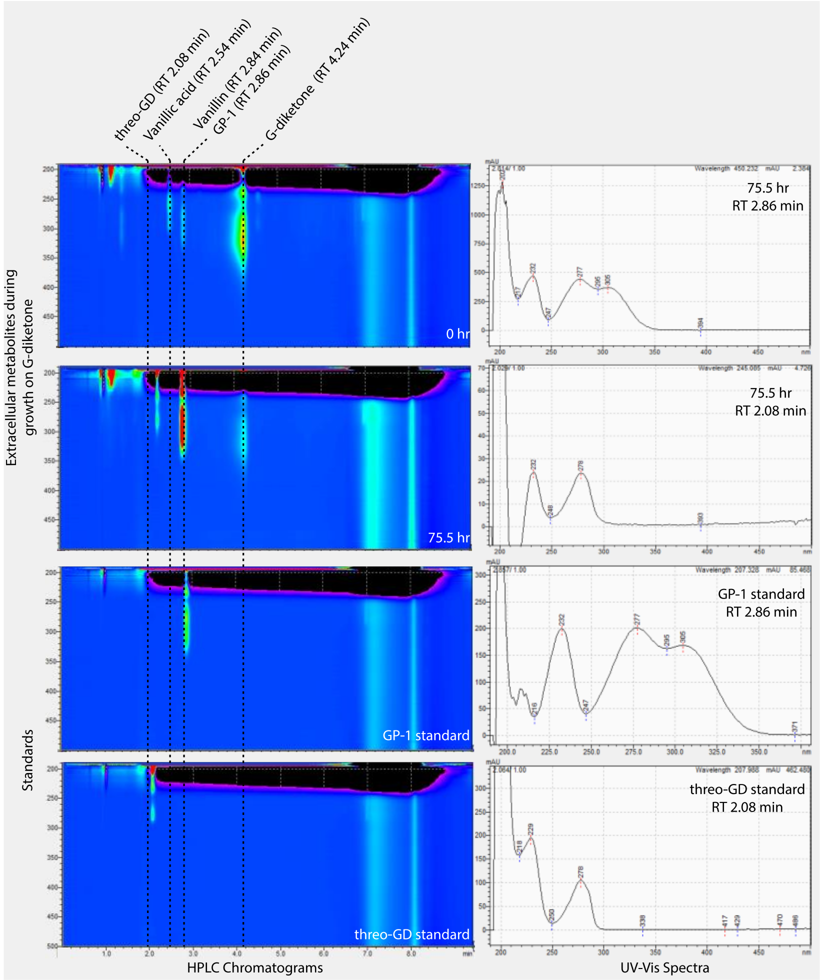

Figure S1. HPLC-UV analysis of extracellular compounds found in the media of cultures grown on glucose plus G-diketone at timepoints 0 hours and 75.5 hours and comparison to standards.

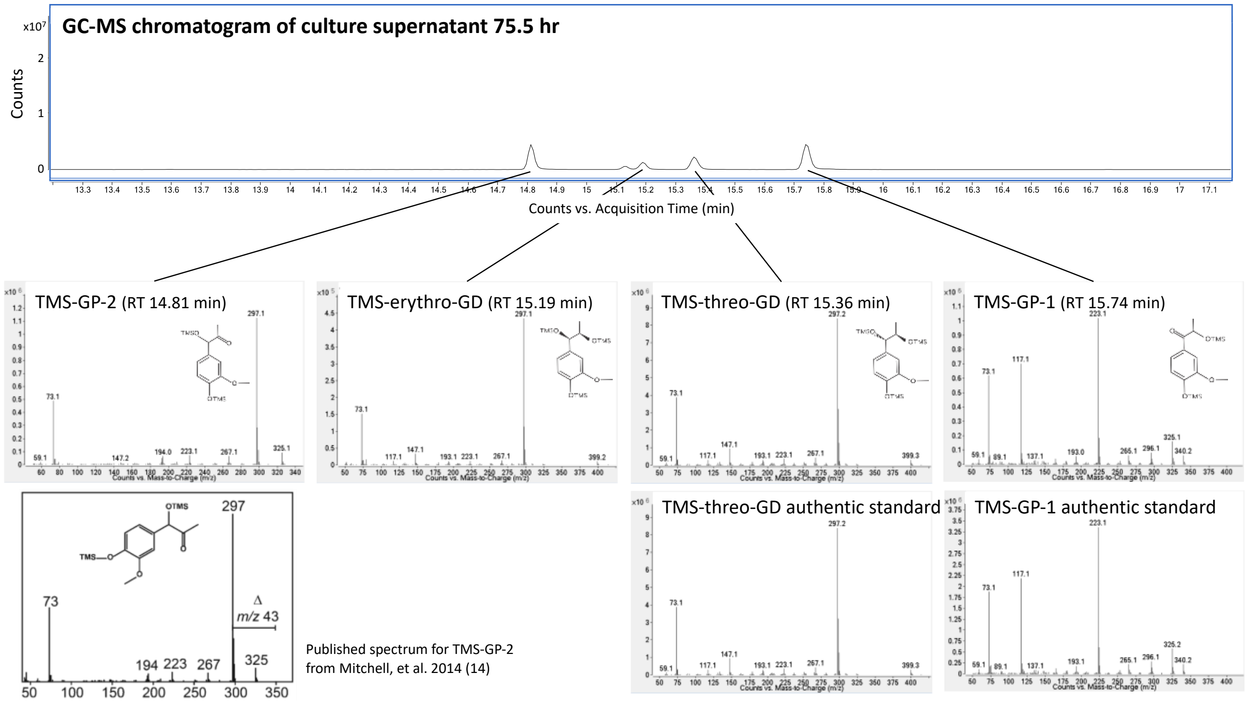

Figure S2. Mass spectra of extracellular compounds identified in the media of cultures grown on glucose plus G-diketone at timepoint 75.5 hours analyzed via GC-MS and comparison to GP-1 and threo-GD standards. We have also included the published spectrum for GP-2 from Mitchell (2014) (1) for reference to our proposed identification of GP-2.

Table S2. Quantification of extracellular vanillic acid and vanillin in cultures grown with the indicated aromatic substrates

Substrates*	Vanillic acid (μM)**	Vanillin (μM)**
**G-diketone plus glucose**	21 ± 1	None detected
**GP-1 plus glucose**	28 ± 4	3 ± 0
**Glucose only**	None detected	None detected

* Substrates were normalized to having 0.5 gCOD/L of the aromatic compound plus 0.5 g COD/L of glucose.

** Reported concentrations are average and standard deviations of six separate cultures after overnight incubation.

Table S3. Quantification of vanillin and vanillic acid in G-diketone and GP-1 preparations

Contaminant/Substrate ratio (HPLC peak area)	G-diketone (custom synthesized)	GP-1 (Key Organics)
**Vanillin**	1:77	None detected
**Vanillic acid**	1:1765	None detected

Dataset S1. RPKM data, differential expression testing, and gene location and annotation information from RNA-Seq analysis of cultures grown in the presence of glucose and G-type aromatics.

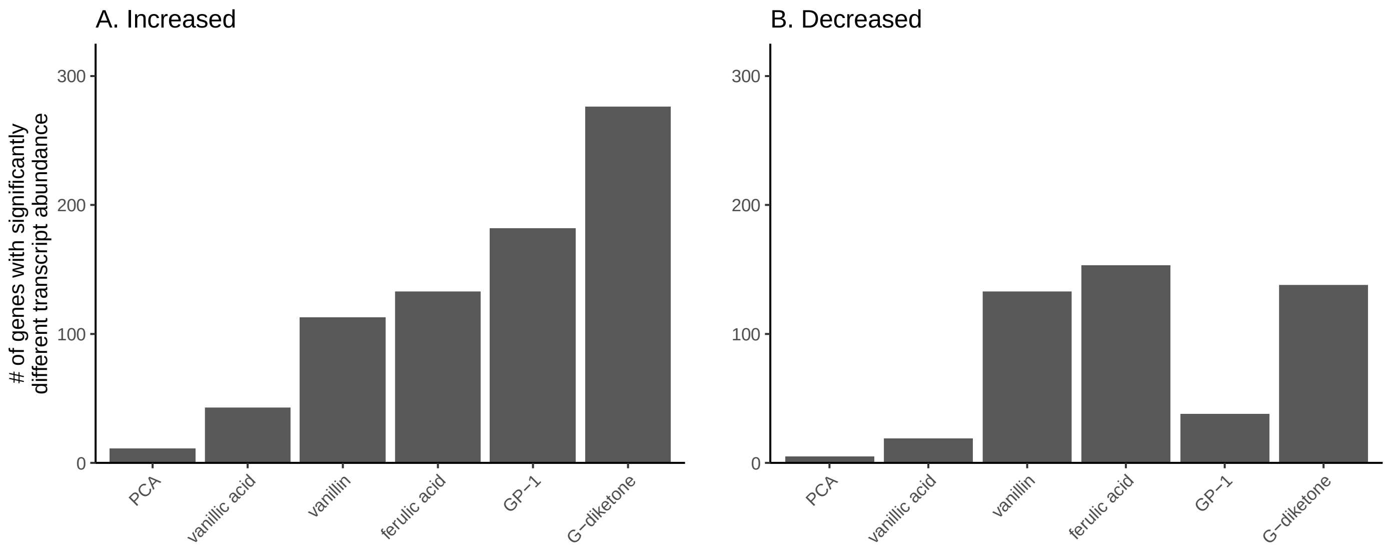

Figure S3. Number of genes that significantly differ in transcript abundance during growth in the presence of glucose alone compared to glucose plus each indicated aromatic substrate. Genes with a q-value < 0.01 are considered significant. Panel A displays counts of genes with increased transcript abundance compared to the glucose control, while Panel B displays counts of genes with decreased transcript abundance.

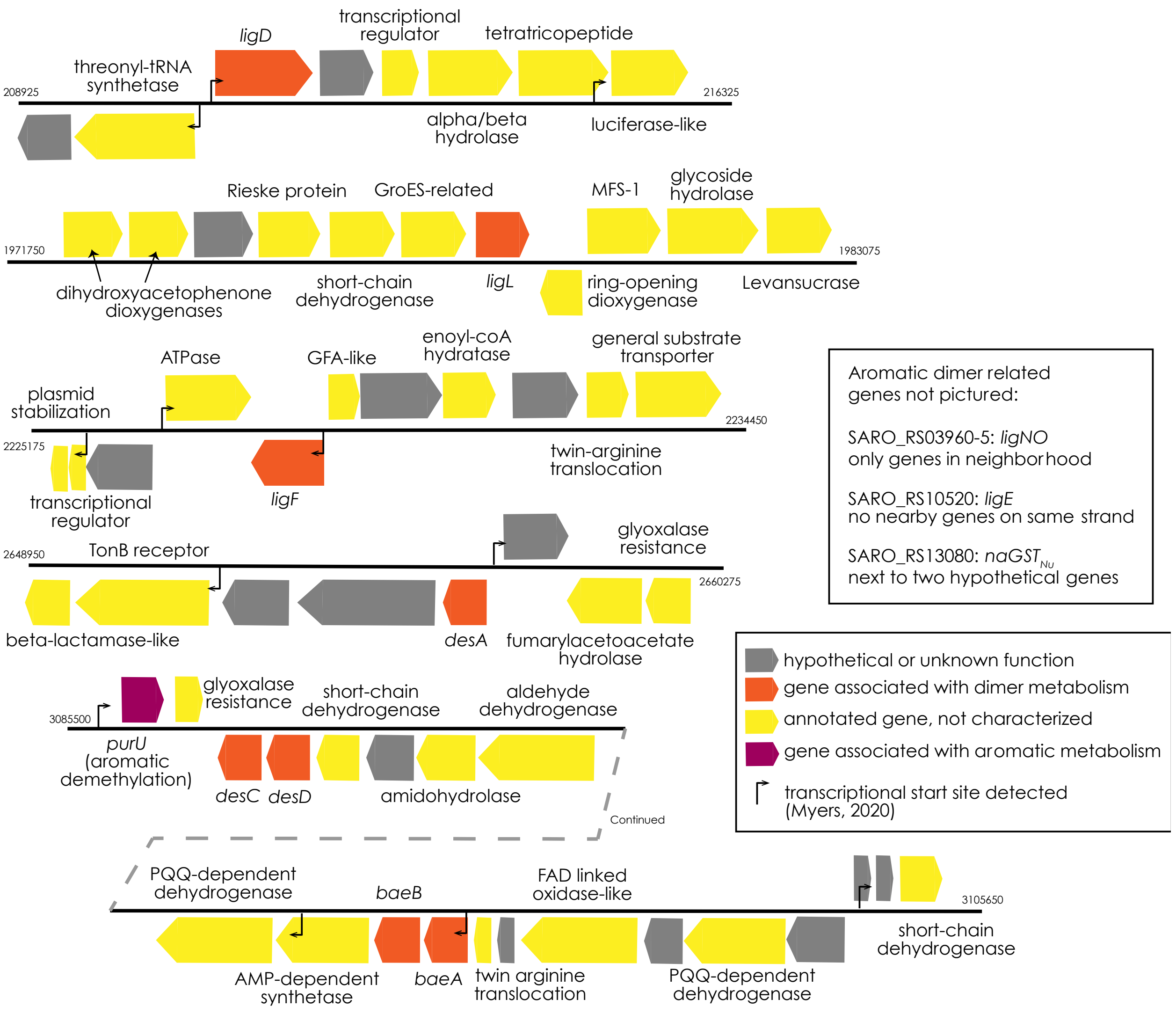

Figure S4. Genomic neighborhoods of *N. aromaticivorans* genes associated with β-O-4 linked aromatic dimer degradation. Shown are position and genes linked to transcripts with increased abundance when cells were grown in the presence of G-diketone and glucose compared to glucose alone.

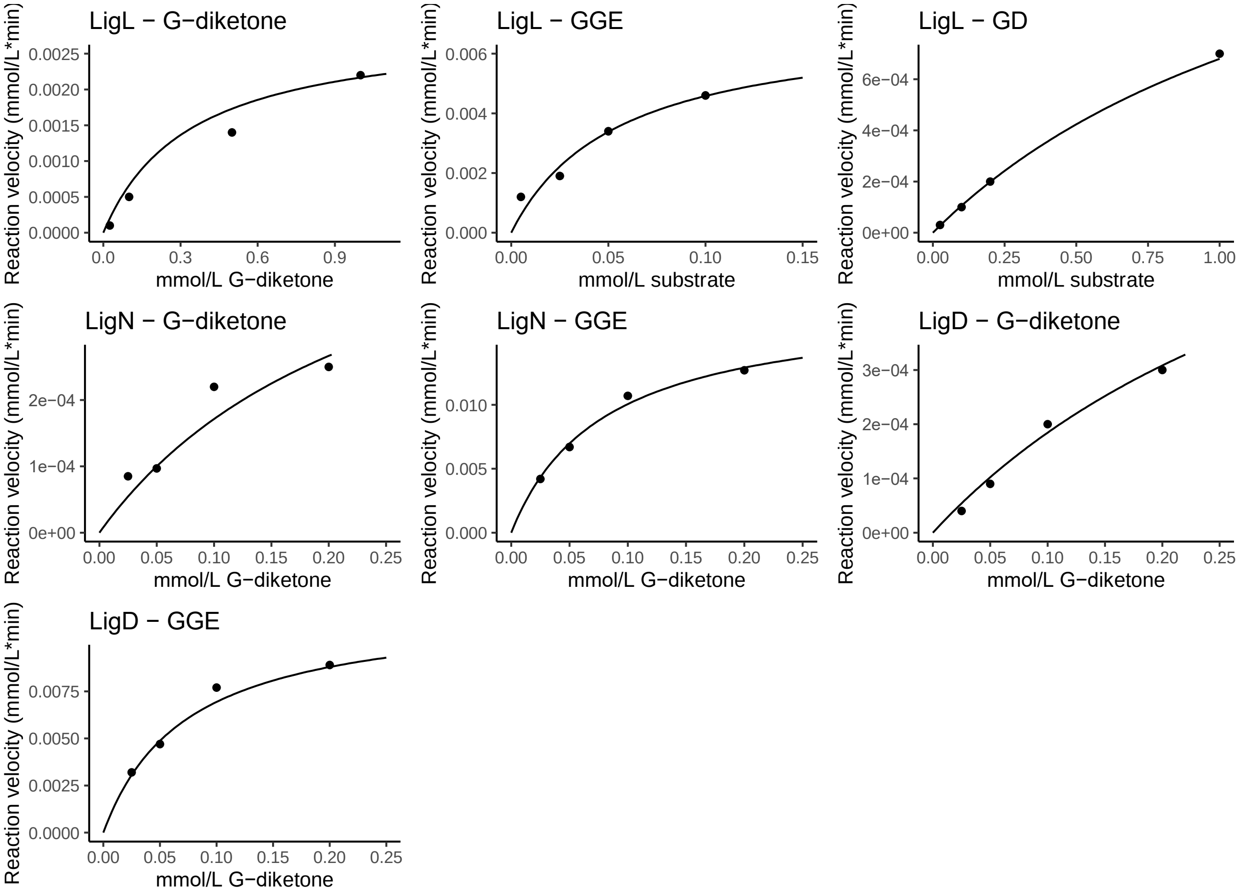

Figure S5. Reaction velocity vs. substrate concentration used to calculate K_m and k_{cat} values for LigL, LigN, and LigD on GGE, G-diketone, and GD.

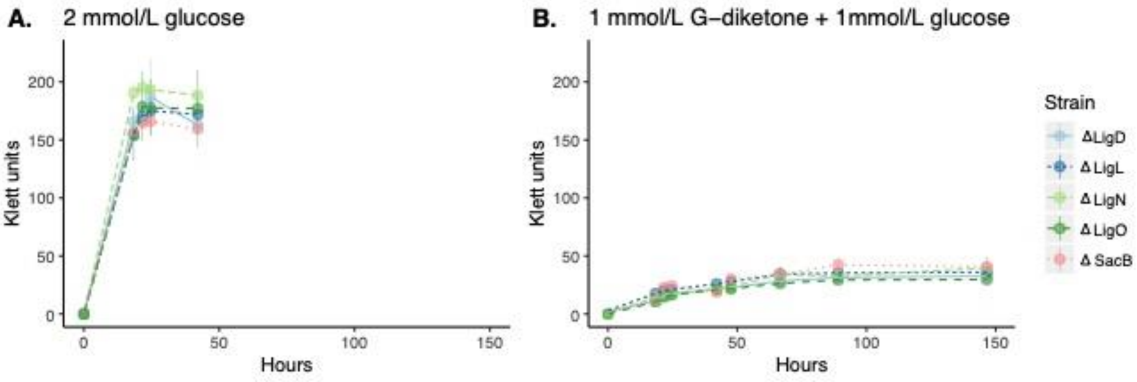

Figure S6. Growth of individual 12444ΔLigLNDO deletion strains on glucose and glucose plus G-diketone compared to that of the 12444ΔSacB parent strain.

Table S4. Multiple-reaction monitoring (MRM) of compounds quantified using HPLC-MS in this study.

Compound	MW (g/mol)	Parent (-) m/z	Transition 1	Transition 2	Transition 3
G-diketone	194.19	193.1	193.1 -> 136.1 CE22	193.1 -> 107.1 CE30	193.1 -> 122.1 CE25
GP-1	196.2	195.2	195.1 -> 180.1 CE15	195.2 -> 136.0 CE22	195.2 -> 108.0 CE25
Vanillic acid	168.15	167	167.0 -> 152.1 CE19	167.0 -> 107.9 CE19	167.0 -> 123.0 CE14

References:

Myers, KS, Vera, JM, Lemmer, KC, Linz, AM, Landick, R, Noguera, DR, Donohue, TJ. 2020. Genome-wide identification of transcription start sites in two Alphaproteobacteria, *Rhodobacter sphaeroides* 2.4. 1 and *Novosphingobium aromaticivorans* DSM 12444. Microbiology Resource Announcements 9(36) e00880-20.
